# The American Otological Society

**Published:** 1888-08

**Authors:** 


### THE AMERICAN OTOLOGICAL SOCIETY.

Twenty-first annual meeting was held at Pequot House, New London, Connecticut, July 17, 1888.

The Society was called to order by the President, Dr. J. S. Prout, of Brooklyn, who alluded in his opening address to the loss sustained by the Society in the death of Dr. C. R. Agnew, of New York, and suggested that action be taken.

On motion, Dr. Gorham Bacon and Dr. W. H. Carmalt were appointed to prepare an appropriate minute on the subject.

Dr. W. H. Carmalt presented the report of the Committee of Conference on the Congress of American Physicians and Surgeons.

The report was accepted, and it was decided that when the Society adjourns it adjourn to meet at the Arlington Hotel, Washington, D. C., September 18, 1888, at 11 a. m.

On motion, it was decided that the meeting should be strictly for scientific matters, and should not be regarded as a business meeting; that it should not take the place of the annual meeting.

Two papers by Dr. S. Sexton, of New York, entitled "Periostitis Externa of the Mastoid," and "Some of the Indications for Excision of the Drumhead and Malleus," were read by title,

A paper was read by Dr. C. H. Burnett, of Philadelphia, entitled "A Case of Aural Vertigo (Menière's Disease) Relieved by Excision of the Membrana Tympani and Malleus." The patient was an unmarried woman, thirty-seven years of age, who six years previous had been under treatment for chronic naso-pharyngeal catarrh, and chronic catarrh of the left middle ear, accompanied with impairment of hearing, tinnitus aurium, and a sense of fullness in the affected organ. Treatment of the catarrhal disease of the ear produced little, if any, benefit. After the lapse of six years, the symptoms already named grew worse, and there was superadded marked aural vertigo. The membrana tympani in the line of the malleus handle was found adherent to the promontory, and the consequent retraction of the entire chain of bones was supposed to be the cause of the aural vertigo and probably of the sense of fullness, and of the tinnitus.

The operation of excision of the membrana tympani and the malleus was performed under ether May 21, with immediate relief to the aural vertigo, which before had often been sufficient to cause the patient to hold to a lamp-post for support, and to the sense of pressure and tinnitus, which good result has been maintained to the present time. The hearing was practically unaffected by the operation. The incus was detached from the stapes, but could not be removed, as it slipped into the attic, and grappling for it is not advisable on account of the risk of irritation.

Its removal, furthermore, would have no effect on the result of the operation.

Dr. S. Sexton then exhibited a new portable battery for the storage of electromotor force, and a new head-lantern for the employment of electric light in surgery. The battery was made at his suggestion. It consists of three cells, and will light a six-candle electric light. The lantern is a modification of that of Trouve, but much lighter, and having a non-conducting base. Such a light is almost necessary in operations on the ear, when ether is used as an anæsthetic. The battery will work continuously for twenty-four hours, and will retain its power for several weeks or months. It may be charged by a dynamo or by twelve gravity cells. When not in use, the storage battery may be kept in connection with the gravity cells.

Dr. A. H. Buck, of New York, read a paper on "Reflex Influences in the Production of Naso-Pharyngeal Catarrh." The object of the paper was to call attention to those comparatively remote exciting causes of naso-pharyngeal catarrh, which act, so far as it is possible to explain their mechanism, through the intervention of the vaso-motor fibres of the sympathetic nerve. Little is known of the direct exciting causes of naso-pharyngeal catarrh. The most common indirect cause is chilling of the surface of the body. According to certain authorities, affections of the teeth should rank next in order of frequency. The author had, however, seen very few cases in which dental disturbance played the part of a promoter of naso-pharyngeal catarrh or of aural disturbance. Some of those indirect causes which he had observed were then enumerated. Irritation of the gastro-intestinal canal is, in not a few instances, a strong exciting cause of naso-pharyngeal catarrh, and of all the aural disturbances growing out of such a catarrh.

Reflex influence, involving the vault of the pharynx and the ear, may emanate from more distant sources.

The author states that he had spoken of these as indirect causes—that is, as factors competent to aggravate a pre-existing but perhaps latent catarrhal disease, but he saw no reason why these reflex influences may not in certain cases play the part of direct exciting causes. He considered it impossible to demonstrate the correctness of this belief, and therefore preferred to adopt the view which assigns to them a less independent rôle.

Dr. Buck then also read "A Contribution to the Anatomy of the Elephant's Ear." The ear was exhibited, and the interesting points indicated. The external auditory canal is embedded in air-containing cells, and is six and a half inches long. The canal at the time of examination was filled with desquamated material. In the middle ear, the handle of the hammer seems to lie in a horizontal plane; it is not vertical, as in the human being. The anterior part of the drum-cavity is completely cut off from the posterior part. The Eustachian tube comes up through a system of air-cells and opens through one of them close to the drum-membrane. Under the floor of the tympanic cavity there are three septa, making stall-like spaces. Two of these are quite long, six or seven inches. The labyrinth and other parts were not exhibited.

A paper by Dr. Huntington Richards, of New York, was then read by Dr. Buck, on "A Case of Polypoid Angioma of The Ear," and one on "A Case of 'False Drum Membrane.'"

Dr. J. B. Emerson, of New York, read a paper on "The Flexible Catheter As a Drainage-Tube—With Cases."

The author cited several cases, exhibiting the use of the flexible catheter as a drainage-tube.

With deeply seated inflammation of the auditory canal or mastoid cells, maintenance of drainage through a fistula is a necessity; and to prevent closure of the fistula, either by granular growth or natural healing, is important.

Dr. Emerson recommends the use of the flexible catheter as generally the best means to employ, and states his reasons to be the comparative comfort and safety, together with convenience of control by both surgeon and patient. The efficiency observed in the use of the flexible catheter was also referred to.

Dr. Charles J. Kipp, of Newark, reported on "Three Cases of Transient Bilateral Horizontal Nystagmus in Connection with Purulent Inflammation of the Middle Ear."

Dr. J. O. Tansley, of New York, exhibited "An Improved Aural Snare."

The snare was devised to overcome an objection to the ordinary Wilde snare—that is, the little jerk and rebound of the instrument when the growth is cut through. The instrument exhibited consists of a small tube through which the wire passes, to be connected with a small bobbin, by the turning of which it is gradually shortened.

A paper by Dr. S. Sexton, of New York, on "Foreign Bodies in the External Auditory Canal," was read by title.

The following officers were elected:

*President*—Dr. J. S. Prout, of Brooklyn.

*Vice-President*—Dr. Gorham Bacon, of New York.

*Secretary and Treasurer*—Dr. J. J. B. Vermyne, of New Bedford, Massachusetts.

*Committee on Membership*—Dr. A. Mathewson, Dr. D. B. St. John Roosa, and Dr. John Green.

*Delegate to Congress of American Physicians and Surgeons*—Dr. W. H. Carmalt, of New Haven; *Alternate*, Dr. G. Bacon, of New York.

The Society then adjourned to meet at the Arlington Hotel, Washington, D. C., Tuesday, September 18, 1888.

---

